# The formation of a medical student research committee and its impact on involvement in departmental research

**DOI:** 10.1080/10872981.2018.1424449

**Published:** 2018-01-18

**Authors:** Stuart Schexnayder, Hunter Starring, Matt Fury, Arthur Mora, Claudia Leonardi, Vinod Dasa

**Affiliations:** a Department of Orthopaedics, Louisiana State University Health Sciences Center, New Orleans, LA, USA; b Harvard Combined Orthopaedic Residency Program, Harvard Medical School, Boston, MA, USA; c School of Public Health and Tropical Medicine, Tulane University, New Orleans, LA, USA

**Keywords:** Medical student, medical school, research, orthopaedics

## Abstract

Over the past ten years, medical students have increased their research activity to be competitive for orthopaedic residency positions throughout the country. This increase may favor students at institutions with a strong history of research production and well-established research departments with supporting staff. To compete with these institutions, a Musculoskeletal Research Committee was developed at a southern academic institution to provide a mutually beneficial link between orthopaedic research faculty and medical students. This manuscript describes the formation of this committee and the resultant involvement of young medical students in departmental research over a one year period. Composed of students and faculty, the committee developed a Research Guide for Medical Students, Research Database and Student List, Medical Students’ Webpage, and Routing Form, and holds quarterly meetings for those students active in orthopaedic research. With this platform, the committee aimed to increase young student involvement in research and provide a stratified level of study participation among upper-level students for continued mentorship. In one calendar year, the total number of first and second-year students participating in department research increased 460% (5 to 28). Also, the total number of research projects with student involvement from these two classes increased 780% (5 to 44). The introduction of a research committee is an effective method of stimulating student interest in departmental research. Early participation results are promising, and this method may be applicable to other departments and institutions hoping to increase research productivity.

**Abbreviations:** IRB: Institutional Review Board

## Introduction

Research is an important aspect of medical training and plays a vital role in the advancement of clinical medicine. It teaches educational skills that are traditionally absent in many medical school curricula but important in building a career founded on evidence-based medicine [1–3]. Research production also serves to improve institutional reputation and visibility while facilitating faculty advancement. To these ends, departments can benefit greatly from medical student involvement in research endeavors. Medical students, on the other hand, seeking to be competitive for orthopaedic residencies, have increased their own research activities significantly over the past ten years. This growing emphasis on research in the application process may stimulate younger students to seek out further research experiences [4]. Some medical schools offer summer programs for first-year students or electives in research to fourth-year students, but these opportunities are likely too brief for students to complete a study or too late to include during the residency application process [5–7].

Medical students may then seek to participate in research opportunities external to the formal curriculum, but face significant barriers including a lack of time, competing educational demands, and unfamiliarity with the research process [8]. Many institutions, particularly those with a strong history of research production, have established research departments with support staff in place to assist faculty, residents, and medical students [9–11]. As the emphasis on research grows across the country, departments that have not historically been active in research are now searching for ways to increase productivity. This paper describes the formation of a medical student research committee in March of 2016 and the resultant involvement of young medical students in departmental research.

With the aid of the departmental Director of Research, a Musculoskeletal Student Research Committee was developed to provide a mutually beneficial link between orthopaedic research faculty and medical students at a southern academic institution. The goal of the committee was to overcome common barriers described previously in order to increase the amount, quality, and interest in research within the department of orthopaedics. Hence the mission statement of the committee was written, ‘To connect students with the information and faculty needed to guide and maximize medical students’ time to choose, start, and complete research of the highest quality.’

## Methods

### Forming the committee

The committee consists of a Committee Director (Orthopaedic Director of Research), Faculty Advisor (Chairman of the Orthopaedic Department), Faculty Coordinator (Research Coordinator of the Orthopaedic Department), Faculty Statistician, and Faculty Project Developer. The medical student roles of Chairman, Senior Advisor to the Chair, and Chair-Elect are filled with third-year, fourth-year, and second-year students, respectively. At the end of the school year, all students ascend in role, and nominations for the new Chair-Elect are put foward by the student committee members and selected by the faculty on the committee. Only students that are currently participating in committee research are eligible for nomination to ensure continued leadership regarding the responsibilities of the position.

### Chairman, senior advisor to the chair, and chair-elect responsibilities

These three students serve as a bridge between the medical students, residents, and faculty. The Chairman’s responsibilities include emailing students, faculty, and the Director of Research to assist in all matters related to research. This consists of managing the Medical Students’ page on the department website, maintaining an online research database of existing and proposed projects, and planning all quarterly meetings – explained in further detail below. The Senior Advisor to the Chair provides counsel to the Chairman and Chair-Elect regarding past research experiences, in addition to serving as a mentor to younger students and a liaison to upper-level medical students. The Chair-Elect assists the Chairman with their tasks and acts as a liaison and visible contact for the first and second-year students who spend most of their time in the lecture halls on campus. A responsibility shared between the committee is to continually update the Research Guide, as detailed below.

### The research guide

Reflecting on the past experiences and difficulties conducting medical research, the committee developed a ‘Step by Step Research Guide for Medical Students’, detailing how to complete institutional training modules, Institutional Review Board (IRB) documentation, and miscellaneous information such as instructions on obtaining data privileges at various hospitals. Furthermore, specific instructions are included to guide students seeking to join fellow medical students on active projects, assist on a resident’s study, or begin their own project under the guidance of a faculty physician. The Research Guide also includes instructions regarding access to the Research Database and Student List.

### Research database and student list

The Research Database and Student List are documents developed and maintained by members of the committee that communicate opportunities for research activities to students. The Research Database contains potential research ideas generated by the orthopaedic faculty, resident projects in development, and ongoing projects in need of student assistance. A second document, the Student List, contains student-run projects only. Projects on this list are closely monitored by the committee to identify roadblocks to progress and provide quick resolution. The Director of Research, along with the Chairman and Chair-Elect, collaborate to ensure these documents are current. Both documents allow students to view up-to-date project ideas, as well as available spots on a variety of studies in the department and are fundamental to the productivity of the committee. These documents are readily accessible to students on the ‘Medical Students’ portion of the departmental website.

### Medical students’ webpage

The Medical Students’ Webpage was created by the committee on the existing Department of Orthopaedics’ research website and can be visited by students. This was developed to provide access, in one setting, to all resources produced by the committee to assist students in research. These resources include the Step by Step Guide, Student List, examples of literature reviews, completed IRB and Institutional Biosafety Committee (IBC) forms, as well as other documents commonly needed to start research. If a student elects to begin a new study, the department required Routing Form can be found on the webpage as well.

### Routing form

The Routing Form, created by the committee, was designed to follow the steps in conducting research. It requires that students complete a literature review, formulate a research question and hypothesis, determine study design, data source, variables of interest, and selection of measurements. The committee also requires assignment of authorship inclusion and order on the form to avoid disagreements upon submission for publication. In order to ensure study progress, students and faculty select target conferences for abstract submissions and other target dates. This form serves as a guide to aid students in the research process, but it also helps to ensure the feasibility and merit of the proposed study by requiring several faculty approvals to proceed.

Upon securing approvals from the faculty advisor and director of research, the faculty project developer and statistician meet with students to refine the proposal and aid in preparation of successful IRB submission.

### Quarterly meetings

The purpose of the quarterly meetings is to discuss new and ongoing projects, educate students in the research process, and provide students with assistance to address common obstacles in research. Preceding these meetings, the committee assembles to set the meeting agenda and discuss any issues with active projects that may require faculty intervention. Also, the Chairman of the committee emails each faculty member to survey for new project ideas to be shared with students. This provides students with a narrower scope of projects aligned with faculty interests to ensure faculty support.

Additionally, each meeting has a presentation given on research training as follows:

Q1. Introduction to Student Webpage, and Instruction on IRB/Literature Review Completion (given by Chairman)

Q2. Research Design and Methodology (given by Faculty Project Developer)

Q3. Data Collection, Analysis, and Basic Biostatistics (given by Faculty Statistician)

Q4. Manuscript Completion (given by Committee Director)

At the end of each meeting, time is reserved for students to address issues with current projects, interact with the Director of Research, and connect with classmates interested in beginning projects. Networking with other students, particularly students in classes above or below, is important as the committee encourages a stratified level of experience on new studies.

### Stratified study participation

The committee determined that an attempt should be made to have representation on projects from each level of training. For example, a new study initiated by two first or second-year students should also seek an upper-level student and resident to be a part of the project. This provides for peer mentorship among the team. Upper-level students and residents can offer greater clinical knowledge and research experience. It is expected that as those first and second-year students advance to upper-level students, they assume the same mentor roles on future projects.

## Results

In one calendar year, from February 2016 to February 2017, the total number of first and second-year medical students (from approximately 200 students per medical school class) participating in department research increased 460% from 5 to 28 (Table 1). Which was an increase from 1.3% to 7% participation in the two classes combined. The total number of projects with student involvement increased 780% from 5 to 44 over this timespan (Table 2). This was calculated as the summation of the number of projects that each individual student had participation in. Whereas there were no students involved in multiple projects in 2016, there were 11 students involved in multiple projects in 2017. Table 3 identifies that there was no difference in study type participation between 2016 and 2017, but participation was primarily in retrospective cohort studies.

**Table 1.** Number of student participants

Student classification	2016	2017	% Change
First year students	1	17	1600%
Second year students	4	11	175%
Total	5	28	460%

**Table 2.** Extent of involvement

Student classification	2016	2017
First year students	*n = 1*	*n = 17*
1 project	1	14
2 projects	-	1
3 projects	-	2
Second year students	*n = 4*	*n = 11*
1 project	4	3
2 projects	-	6
3 projects	-	1
4 projects	-	1
Total projects involving students	5	44

**Table 3.** Study type stratification.

Student classification	2016	2017
First year student projects	*n = 1*	*n = 22*
Retrospective cohort	1	20
Prospective cohort	-	2
Second year student projects	*n = 4*	*n = 22*
Retrospective cohort	3	19
Prospective cohort	1	3

## Discussion

### Student involvement

In one year, there was a notable increase in research participation among early-year medical students, as indicated by the number of students as well as the total number of projects with student involvement. Although this study did not directly survey medical students regarding their motivations behind increased research participation, the authors postulate that the structure, availability, and creation of resources removed barriers, provided guidance, and created a framework in which motivated students could thrive. The quarterly meetings create a curriculum of learning research skills while providing mentorship for first-year students by more senior students. This support structure may also enable young students to be aggressive in their research endeavors and participate in multiple projects – in 2017, one student was involved in as many as four projects.

The benefits of increasing medical student research participation may extend further than the Department of Orthopaedics. As more students gain a greater understanding of the research process and the importance of these efforts, they may utilize these skills to make an impact in other fields and bolster the overall academic contributions of the university. After all, evidence-based medicine is the gold standard of practice in all fields of medicine [12,13]. At a minimum, the committee gives students exposure and understanding of clinical research, which is vital in training future physicians in interpreting and applying research to everyday practice.

### Medical student focus

The Musculoskeletal Research Committee is a dynamic group centered on student research issues. Before the committee’s existence, students traditionally reached out to the Director of Research with issues, and then scheduled a meeting to discuss starting the research process as well as project ideas. This was burdensome to the Director because of his clinical and educational schedule and normally required multiple meetings before the official start of the project. With the development of the committee, the common research inquiries are directed to the Chairman and Chair-Elect, and answers are outsourced to the student group. This system also informs the Chairman and Chair-Elect of common questions that should be addressed at the quarterly meetings. Having a student-directed line of communication offloads the demand on faculty and improves the pace of communication. Any questions the Chair and Chair-Elect cannot answer can then be forwarded directly to the Director of Research. This shortens the response time and provides more effective communication. In addition, the quarterly meetings provide students with a scheduled opportunity to interact and ask questions to the Director of Research in a face-to-face setting.

### Faculty and medical student benefit

The committee provides a mutually beneficial relationship that allows students access to projects and assists faculty in finding students interested in participation. Through committee successes regarding the dissemination of research education and resources to students, medical students are provided with research tools that may enhance their contribution to projects. The same benefits can be applied to residents. Since the ACGME requires an orthopaedic resident to complete a study worthy of publication in a respected academic journal, a research partnership with co-investigators who have relatively fewer time demands is a desirable situation[14]. Furthermore, students may benefit from resident mentorship on topics related to medical school, the match process, and residency. Early relationships formed between students and faculty may ease the awkward approach by students for a letter of recommendation and provide faculty with more substance to include in the letter-writing process. In addition, the research experience provided by the committee is beneficial to students who pursue medical specialties outside of orthopedic surgery as they participate in a collaborative environment, practice research skills, and, at the very least, learn about topics related to the musculoskeletal system – the most common reason that patients present for medical care [15,16].

### Continued improvements

It is recognized that continued modification is necessary for improvement. The research guide developed by the committee has thus far been successful in prompting more students to begin research, but undergoes continuous revision and is far from comprehensive. As the number of students involved in research grows, new complications will arise requiring new solutions.

As stated previously, one committee goal is to provide mentorship to first and second-year students’ studies by emphasizing third and fourth-year student participation in projects. While the existence of the committee is still young, it is anticipated that the benefit of study member stratification will grow as the current first and second-year students, now with formal training in research, have progressed to senior students. Although the majority of students are participating in or leading retrospective studies as the demand for publications on applications continues to rise [4], more emphasis will be placed on guiding first and second-year medical students toward performing prospective studies as they are learning and beginning the research process earlier in medical school.

It is possible that the increased participation may have arisen due to increased interest in orthopaedics from class to class. However, the difference in first-year participation was substantial and previous studies describe an indecisiveness most first-year medical students have toward selecting a specialty of interest due to their lack of clinical exposure [17–20]. Furthermore, the committee has fostered a resource for general research participation that welcomes and encourages all students, including those whose medical interests lie elsewhere.

The committee has received praise from the student participants in studies before and after the development of this committee for providing a platform that addressed the difficulties they faced in conducting research effectively. Therefore, it is believed that all students may value the guidance and opportunities offered by this committee. This progress has been noted across the local medical school campus – the Student Government Association has requested a presentation by the committee to encourage replication of the committee in additional fields of academic medicine, since a research model such as this has never been implemented. This year-long experience has demonstrated that students are willing to make the time and have the desire to participate in research. While many faculty possess the same desire, this committee provides the structure to connect and benefit both parties. Although data regarding medical student perceptions was not formally collected during the first year of implementation, the purpose of this paper was to provide a guide for medical schools and residency programs to establish similar committees to increase research participation at their institutions. The authors will continue to assess the long-term effect on research productivity. Although this committee structure may not be applicable to all academic settings, the core principles within – prioritization of mentorship and collaboration, along with the establishment of clear and effective lines of communication – are universally beneficial.

The amount of research experiences and publications that medical students are participating in to match into competitive residency programs continues to increase. From 2007 to 2014, the mean number of research experiences for successful orthopaedic applicants increased from 2.6 to 3.7. The number of publications, abstracts, and presentations increased from 3.0 to 6.7 during that time frame as well [4]. To build a competitive curriculum vitae, a significant portion of medical students take a year off from school in order to pursue research. In fact, of the students that take a year off in medical school that are interested in matching into a highly competitive specialty, 59% did so to increase their competitiveness of their residency application [21]. Not only does this decision extend the educational process, it sacrifices a year of peak earnings for future physicians. Since research is becoming much more imperative on residency applications in fields other than orthopaedics [22], this committee model can be implemented by students and faculty in any medical academic specialty. Regardless if students continue to perform research as clinicians, the understanding and interpretation of research is vital to practicing a career of evidence-based medicine. This manuscript demonstrates how the formation of a student committee, along with supportive department faculty, may quickly grow the research environment at institutions seeking to improve their research activity, and how this partnership may benefit students, residents, faculty, and the medical profession as a whole.

## References

[1] Seymour E, Hunter A, Saursen SL, et al Establishing the benefits of research experiences for undergraduates in the sciences: first findings from a three-year study. Sci Educ. 2004;88(4):493–6.

[2] Russell SH, Hancock MP, McCullough J. The pipeline: benefits of undergraduate research experience. Science. 2007;316(5824):548–549. 17463273 10.1126/science.1140384

[3] Willison J, O’Regan K Commonly known, commonly not known, totally unkown: a framework for students becoming researchers. Higher Educ Res Dev. 2007;26(4):393.

[4] DePasse MJ, Palumbo MA, Eberson CP, et al Academic characteristics of orthopaedic surgery residency applicants from 2007 to 2014. Bone Jt Surg. 2016;98:788–795. 10.2106/JBJS.15.00222 27147692

[5] Elnicki MD, Gallagher S, Willet L, et al Course offerings in the fourth year of medical school: how U.S. medical schools are preparing students for internship. Acad Med. 2015;90(10):1324–1330. 27002885 10.1097/ACM.0000000000000796

[6] Yale School of Medicine Summer research. [updated 2017; cited 1030 2017]. Available from: https://medicine.yale.edu/education/research/student/fundingop/summerresearch.aspx.

[7] The Johns Hopkins University Summer internship program. Johns Hopkins Medicine Web site. [cited 2017 10 30]. Available from: https://www.hopkinsmedicine.org/education/graduate-programs/student-life/diversity/sip.html.

[8] Metcalfe D Involving medical studnets in research. J R Soc Med. 2008;101(3):102–103. 18344462 10.1258/jrsm.2008.070393 PMC2270240

[9] Harvard Medical School Research administration. [updated 2017; cited 1030 2017]. Available from: https://hms.harvard.edu/research/hms-researcher-resources/research-administration.

[10] The Johns Hopkins University Research programs and opportunities. Johns Hopkins Medicine Web site. [updated 2017; cited 1030 2017]. Available from: https://www.hopkinsmedicine.org/som/students/research/index.html.

[11] Georgetown University School of Medicine Research opportunities for medical students. GU School of Medicine Web site. [updated 2017; cited 1030 2017]. Available from: https://som.georgetown.edu/research/students#Faculty Members.

[12] Melnyk BM, Fineout-Overholt E, Giggleman M, et al A test of the ARCC model improves implementation of evidence-based practice, healthcare culture, and patient outcomes. Worldviews Evid Based Nurs. 2017;14(1):5–9. 28002651 10.1111/wvn.12188

[13] Djulbegovic BGG Progress in evidence-based medicine: a quarter century on. Lancet. 2017;390(10092):415–423. 28215660 10.1016/S0140-6736(16)31592-6

[14] Accreditation Council for Graduate Medical Education ACGME common program requirements. rule II.B.5.b). (2). 2017.

[15] Rekola KE, Keinanen-Kiukaanniemi S, Takala J Use of primary health services in sparsely populated country districts by patient with musculoskeletal symptoms: consultations with a physician. J Epidemiol Community Health. 1993;47(2):153–157. 8326275 10.1136/jech.47.2.153 PMC1059745

[16] Kelsey JL, White AA 3rd, Pastides H, et al The impact of musculoskeletal disroders on the population of the USA. J Bone Joint Surg Am. 1979;61(7):959–964. 158597

[17] Babbott D, Baldwin DC Jr, Jolly P, et al The stability of early specialty preferences among US medical school graduates in 1983. JAMA. 1988;259(13):1970–1975. 3346978

[18] Compton MT, Frank E, Elon L, et al Changes in U.S. medical students’ specialty interests over the course of medical school. J Gen Intern Med. 2008;23(7):1095–1100. 18612751 10.1007/s11606-008-0579-z PMC2517937

[19] Kassebaum DGSP Medical students’ career indecision and specialty rejection: roads not taken. Acad Med. 1995;70(10):937–943. 7575926 10.1097/00001888-199510000-00018

[20] Markert RJ Change in specialty choice during medical school. J Fam Pract. 1983;17(2):295–300. 6875487

[21] Pathipathi AS, Taleghani N Research in medical school: a survery evaluating why medical students take research years. Cureus. 2016;8(8):e741. 27672532 10.7759/cureus.741 PMC5026499

[22] National Resident Matching Program Charting outcomes in the match for U.S. allopathic seniors: characteristics of U.S. allopathic seniors who matched to their preferred specialty in the 2016 main residency match. Washington, DC: National Resident Matching Program; 2016.

